# Factors associated with self-reported non-completion of the hepatitis B vaccine series in men who have sex with men in Brazil

**DOI:** 10.1186/s12879-019-3970-y

**Published:** 2019-04-23

**Authors:** Artur Acelino Francisco Luz Nunes Queiroz, Álvaro Francisco Lopes de Sousa, Matheus Costa Brandão Matos, Telma Maria Evangelista de Araújo, Sandra Brignol, Renata Karina Reis, Elucir Gir, Maria Eliete Batista Moura

**Affiliations:** 1 Ribeirão Preto College of Nursing, University of São Paulo, Campus Universitário, Avenida dos Bandeirantes, 3900, Bairro Monte Alegre, Ribeirão Preto, SP 14040-902 Brazil; 2 Global Health and Tropical Medicine, GHTM, Instituto de Higiene e Medicina Tropical, IHMT, Universidade Nova de Lisboa, UNL, Lisbon, Portugal; 3 Federal University of Piauí, Teresina, Brazil; 4 Collective Health Institute, Fluminense Federal University, Niterói, Brazil

**Keywords:** Hepatitis B, Vaccines, Men who have sex with men, Social networks, Vulnerabilities, Geosocial networking phone applications

## Abstract

**Background:**

The objective of the present study was to analyze the factors associated with non-completion of the hepatitis B vaccine series among men who have sex with men and use geosocial dating apps in Brazil.

**Methods:**

This was a cross-sectional, population survey-based, analytical study, conducted exclusively online in all the regions of Brazil, with a sample of 1855 men who have sex with men. The data was collected between November 2016 and February 2017, using the social networking website Facebook.

**Results:**

Univariate, bivariate and multivariate analyses showed that 4.7% of the participants reported receiving one dose of the vaccine, 12.5% two doses, 19.4% three doses, and 45.8% did not know. Multivariate analysis showed that level of education (OR = 0.31; CI 95% 0.14–0.72; *p* = 0.007), identification as bisexual (OR = 0.6; CI 95% 0.38–0.95; *p* = 0.030), HIV serological status (OR:2.3; CI 95% 1.58–3.34; *p* = < 0.001) and frequency of access to health services (OR = 2.38; CI 95% 1.53–3.72; *p* = < 0.001) were associated with not completing the vaccine series. Low completion of the hepatitis B vaccine series was detected in the population studied.

**Conclusion:**

Completion of the hepatitis B vaccine series was low among men who have sex with men and use geosocial dating apps in Brazil. The factors associated with non-completion were related to social, individual and healthcare (programmatic) vulnerabilities.

## Background

Infection by the hepatitis B virus (HBV) continues to be a major health problem. Its estimated prevalence is two billion people worldwide, with associated subclinical and clinical manifestations. Among those who are infected, around 240 million are chronically infected and have a higher risk of incurring serious hepatitis-related complications, such as cirrhosis and hepatocellular carcinoma [1].

The hepatitis B virus is transmitted in two ways: vertically (mother-child transmission) and horizontally, through exposure to contaminated blood or other body fluids, especially through unprotected sexual relations, which is the most common form of exposure [2]. Among the population groups considered highly vulnerable to HBV, men who have sex with men (MSM) stand out for their high prevalence of hepatitis B (15.4%), according to a population survey conducted with this group in Brazil [3].

The vulnerability of MSM to the virus is associated with a higher frequency of several factors, such as engaging in unprotected anal sex, multiple partners, a history of sexually transmitted diseases (STDs), stigma, violence, and difficulty accessing health services [3, 4].

In Brazil, the primary method for prevention is vaccination, which has been provided since the end of the 1990s for children and higher-vulnerability groups. At present, it is universal, through a vaccination series based on three doses. All the vaccination programs focus on the administration of the complete series of doses. However, protective antibody levels are developed after one dose of the vaccine in 30 to 50% of those who are vaccinated, and in 75% after two doses [5, 6].

Although free protection against the virus is ensured at the primary health level, difficulties accessing health services may contribute to low or even incomplete vaccination coverage, especially among vulnerable populations [7]. Based on this scenario, and also in view of the scarcity of Brazilian studies that address HBV immunization in the MSM population, the aim of the present study was to analyze the factors associated with non-completion of the hepatitis B vaccine series among men who have sex with men and use geosocial dating apps in Brazil.

## Methods

This study is part of a multicenter study entitled “Behaviors, practices, and vulnerabilities among men who have sex with men and use geolocation (geosocial) dating apps in Brazil.” It encompassed all of Brazil’s regions and sought to examine the behaviors and practices of men who have sex with men and use geosocial dating apps, and their vulnerability to HIV, hepatitis B and C, and syphilis.

This was a cross-sectional, epidemiological, survey-based, analytical study, since it involved a large contingent of MSM from various states in Brazil, which enabled obtaining primary information on the health status of this group. The study was conducted among MSM who use geosocial dating apps for sexual encounters. The data was collected exclusively online, which promoted the participation of MSM from cities of different sizes and those further away from large urban centers, unlike large epidemiological studies that have previously been carried out with MSM in Brazil, but which have focused on large urban centers and state capitals [8].

### Sample

The sample was calculated based on the estimated MSM population (3.5% of the overall population of men in Brazil [9]) – an estimate widely used in surveys with this population. The sample size calculation took into consideration a maximum permissible error of 5% and a significance level of 5% in the event of a decision to reject the main hypotheses of the analysis [10], and the final sample was fixed in 1855 participants.

### Study protocol

The data was collected between November 2016 and February 2017, using only the Facebook social networking website to publicize the survey. A fixed post was written on a Facebook page *(*https://www.facebook.com/taafimdeque/), which was administered by the two researchers responsible for the data collection and provided not only information on the research but also information related to STDs. Only this fixed post with a link to the study was boosted by the researchers. The post also had an invitation for people to participate and it was continually boosted (weekly) to have access to all of Brazil’s regions, which made it possible to reach the necessary sample size to meet the study objectives. The use of the boost feature is a valuable tool for reaching difficult-to-access populations, since it enables highlighting online content or pages. This type of approach attracts the attention of users and increases the number of views.

The post was accompanied by a hyperlink that provided access to the survey questionnaire, with obligatory and optional questions, which was subdivided into four sections in order to obtain social, demographic and health information on the participants: (1) Personal characteristics; (2) Sociocultural characteristics; (3) Health issues; and (4) Sexual practices. The system was designed so that it would only be possible to move to a new section only if all the obligatory question, selected by the researchers, had been answered; this way, the questionnaire would only be tallied at the end if all the conditions had been satisfied. Therefore, incomplete questionnaires were not saved. To respond to the questionnaire, the participants needed to provide their email, to avoid duplication of responses. The inclusion criteria were: identifying oneself as a cisgender man; being 18 years of age or older; having sexual relation with another man in the last 12 months; and having used apps for sexual encounters at least once in the last 30 days. Users not residing in Brazil were excluded.

The description of the outcome (hepatitis B vaccine history) was self-reported by the participants, since the researchers did not have access to vaccine dose records through immunization booklets; however, the participants were instructed to check their booklets before answering. A person was considered vaccinated if the series (three doses) had been completed and recorded in the immunization booklet; not completely vaccinated if they had received “one or two doses”; and not vaccinated in the case of “zero doses” or the “I don’t know” response option, as per recommendations from the Brazilian Ministry of Health [11].

For analyses in the logistical model, the information was regrouped into a) complete series - participants who reported receiving three vaccine doses; and b) those who had received less than three doses (one or two doses) or did not know how many doses they had received, defined as an incomplete series, according to the definitions established by the Brazilian Ministry of Health [11].

### Data analysis

The data was entered on a Microsoft Excel for Windows worksheet and imported afterward into the data analysis program. The Kolmogorov-Smirnov test was used to assess the normality distribution of the numerical variables (age, income, and length of time of relationship). To describe the factors of interest, descriptive analyses were performed for both the numerical variables and the categories.

For univariate analysis and analysis of the association between immunization status and categorized vulnerability factors, the chi-square test and Fisher’s exact test were used, with a significance level of 0.05. To build the confidence intervals, a confidence of 95% was used, with the help of the Statistical Package for the Social Sciences (SPSS) IBM® Version 23.0.

Univariate and multivariate logistic regression generated the Odds Ratio (OR) and adjusted OR, indicating the odds of non-completion of the hepatitis B vaccine series. The reference categories were: elementary education for education; heterosexual for sexual orientation; HIV positive for serological status; seeking information from health professionals; seeking information online; and higher frequency of use of health services. This made it possible to verify the odds of negative events such as risk factors and, as protectors, the factors that lead to better care and, thus, less likelihood of not completing the hepatitis B vaccine series.

### Ethical aspects

The study was approved by the Research Ethics Committee (Opinion No. 1523003) and strictly complied with all the ethical precepts in Resolution 466/12, with guidelines for research with human subjects in Brazil. The participants read the free and informed consent form online and then signed it, thereby indicating their agreement with the proposed objectives and willingness to participate in the study. This consent was applied and obtained online.

## Results

Among the participants (*n* = 1855), 4.7% reported having received one dose, 12.5% two doses and 19.4% three doses, thus, 36,6% of participants report some type of protection. Another 17.6% reported not having been vaccinated (zero doses), and 45.8% did not know how many doses they had received, as shown in Table 1.

**Table 1:** Sociodemographic characteristics of men who have sex with men and use geosocial dating apps in Brazil. (*n* = 1855)

Factor of interest/vulnerability	N	%	Mean	SD	CI (95%)	Min-Max
Age (in years)			25.74	7.76	(21.0–21.8)	18–90
18 to 20	522	23.2				
21 to 25	836	37.1				
26 to 30	488	21.7				
Over 30	376	16.7				
Education						
Elementary Education	57	2.5				
Secondary Education	445	19.7				
University	1303	57.8				
Postgraduate	445	19.7				
Region of residence						
North	108	4.8				
Northeast	336	14.9				
Center-West	202	9				
Southeast	1133	50.3				
South	424	18.8				
Marital Status						
Single	1696	75.2				
Living as married	516	22.9				
Separated/divorced/widowed	38	1.7				

The average age of the participants was 25.7 years old, with a standard deviation of 7.8 years. The majority had completed their higher education (57.8%); did not practice any religion (63.9%); lived with their parents (55.5%); were single (75.2%); and engaged in casual sexual relationships (49.3%). Majority of the participants lived in the southeast region of Brazil (50.3%) (Table 1).

Table 2 presents the bivariate analysis of the factors associated with completion of the hepatitis B vaccine series among the study participants. In this analysis, association with the study outcome was statistically significant for the following factors: education (*p* < 0.001); Brazilian region of residence (*p* = 0.023); sexual orientation (*p* < 0.001); marital status (*p* = 0.011); presence of STDs (*p* = 0.003); HIV status (*p* < 0.001); use of alcohol during sex (*p* = 0.003); and frequency of use of health services (*p* < 0.001).

**Table 2:** Bivariate analysis of factors associated with completion of the vaccine series for men who have sex with men and use geosocial dating apps in Brazil (*n* = 1855)

Variables	Complete Series						
	Yes		No		Total		*p*-value*
	n	(%)	n	(%)	n	(%)	
Education							< 0.001*
Secondary Education	46	13.2	303	86.8	349	100	
University	231	21.3	856	78.7	1087	100	
Postgraduate	152	59.0	219	41.0	371	100	
Region of residence†							0.023*
North	12	14.5	71	85.5	83	100	
Northeast	59	21.9	211	78.1	270	100	
Center-West	56	31.5	122	68.5	178	100	
Southeast	207	22.4	717	77.6	924	100	
South	95	26.4	265	73.6	360	100	
Sexual Orientation†							< 0.001**
Heterosexual	25	14.3	148	85.7	173	100	
Homosexual	368	26.0	1047	74.0	1415	100	
Bisexual	43	16.5	217	83.4	260	100	
Marital Status							0.005*
Single	320	22.8	1084	77.2	1404	100	
Stable relationship	117	25.9	334	74.1	451	100	
Had an STD in the last 12 months							0.003*
Yes	66	29.6	157	70.4	223	100	
No	352	23.5	1143	76.5	1495	100	
I don’t know	19	13.9	118	86.1	137	100	
HIV status							< 0.001*
HIV+	71	47.7	78	52.3	149	100	
HIV-	304	24.9	918	75.1	1222	100	
I don’t know	62	12.8	422	87.2	484	100	
Prevention via condom***							0.097
Yes	405	23.5	1315	76.5	1720	100	
No	32	23.7	103	76.3	135	100	
Use of alcohol during sex***†							0.003*
Yes	205	24.0	648	76.0	853	100	
No	229	23.0	766	77.0	995	100	
Use of illicit drugs during sex***†							0.338
Yes	94	22.8	318	77.2	412	100	
No	318	77.2	1100	76.2	1418	100	
Frequency of visits to health services							< 0.001*
Monthly	68	31.8	146	68.2	214	100	
2 months	68	27.9	176	72.1	244	100	
6 months	163	26.9	442	73.1	605	100	
Annually	87	24.3	271	75.7	358	100	
Rarely	51	11.8	383	88.2	434	100	
Health information source							
Health professional							< 0.001*
Yes	309	28.6	770	71.4	1079	100	
No	128	16.5	648	83.5	776	100	
Family member							0.515
Yes	24	21.1	90	78.9	114	100	
No	413	23.7	1328	76.3	1741	100	
Television							0.032*
Yes	19	15.6	103	84.4	122	100	
No	418	24.1	1315	75.9	1733	100	
Internet							< 0.001*
Yes	339	79.0	1272	79.0	1611	100	
No	98	40.2	146	59.8	244	100	
Testing and counseling center							< 0.001*
Yes	117	30.9	262	69.1	379	100	
No	320	21.7	1156	78.3	1476	100	
Testing location							0.006*
Yes	61	31.4	133	68.6	194	100	
No	376	22.6	1285	77.4	1661	100	

*Statistical significance was set at 0.05; **Fisher’s exact test; ***30 days prior to the survey

*†Value of n differs due to it not being required to answer the question*

Multivariate analysis (Table 3) indicated that the odds of non-completion of the hepatitis B vaccine series were high among those with less than university level education: those with secondary education (OR = 3.8; CI 95% = 2.6–5.7), or less (OR = 3.2; CI 95% = 1.4–7.4), had the worst outcomes. In relation to HIV serological status, being HIV negative (OR = 2.3; CI 95% = 1.6–3.3) and not knowing one’s status (OR = 3.7; CI 95% = 2.3–5.8) increased the likelihood of an incomplete vaccine series. In terms of access to health information and services, not seeking information from health professionals increased the probability of an incomplete vaccine series (OR = 1.5: CI 95% = 1.1–1.9), while searching for another source of information other than the internet proved to be a protection factor for the occurrence of an incomplete vaccine series (OR = 0.4; CI 95% = 0.3–0.6). Another result worth noting was related to seeking health services; the lower the frequency, the higher the odds of an incomplete vaccine series; the worst scenario was those who rarely seek health services (OR = 2.38; CI 95% = 1.53–3.72). Sexual identity was retained in the model, even though it had no statistical significance, since it improved adjustment quality in the presence of other factors, and also indicated higher protection in the bisexual category (OR = 0.6; CI 95% = 0.4–1.0) (Table 3).

**Table 3:** Multivariate analysis of factors associated with completion of the vaccine series for men who have sex with men and use geosocial dating apps in Brazil (*n* = 1855)

Factor analyzed	Crude OR	CI (95%)*	Adjusted OR	CI (95%)*
Education				
Postgraduate	1.0		1.0	
University	2.6	(2.0–3.3)	2.4	(1.9–3.2)
Secondary Education	4.6	(3.2–6.6)	3.8	(2.6–5.7)
Elementary Education	3.5	(1.6–7.6)	3.2	(1.4–7.4)
Sexual Orientation				
Heterosexual	1.0		1.0	
Homosexual	0.8	(0.5–1.5)	0.8	(0.5–1.5)
Bisexual	0.6	(4.1–9.4)	0.6	(0.3–0.9)
HIV status				
HIV+	1.0		1.0	
HIV-	2.7	(1.9–3.9)	2.3	(1.6–3.3)
I don’t know	6.2	(4.1–9.4)	3.7	(2.3–5.8)
Health information source				
Health professional				
Yes	1.0		1.0	
No	2.0	(1.6–2.6)	1.5	(1.1–1.9)
Internet				
Yes	1.0		1.0	
No	0.4	(0.3–0.5)	0.4	(0.3–0.6)
Frequency of visits to health services				
Monthly	1.0		1.0	
2 months	1.2	(0.8–1.8)	1.4	(0.9–2.2)
6 months	1.3	(0.9–1.8)	1.4	(1.0–2.0)
Annually	1.5	(1.0–2.1)	1.3	(0.9–1.9)
Rarely	3.5	(2.3–5.3)	2.4	(1.5–3.7)

*Confidence Interval (95%)

## Discussion

Self-reported completion of the hepatitis B vaccine series was low among MSM who participated in the study (19.4%), compared to the number of third doses accumulated in the Brazilian adult population, which was 49.3% according to recent official data [12]. These low rates are cause for concern because they occurred in a population with higher exposure to risk factors for hepatitis B virus infection [12]. Thus, this situation seems to worsen in the case of vulnerable populations, such as MSM. The findings of our study indicated that most were not fully vaccinated, were unable to prove vaccination or were unaware of their vaccination status. These percentages were also lower than the vaccination rates reported in other studies conducted exclusively with MSM, which varied between 50.7% [13] and 77.4% [14], even with self-reporting [13] as the data collection method.

However, our results should be interpreted considering the Brazilian context that, unlike other countries, offers the hepatitis B vaccine free of charges to children and adolescents since the beginning of the 2000s [15]. Thus, part of our sample, aged between 18 and 19 years, may have been vaccinated (or started the vaccination scheme) by their parents and do not have this information.

The introduction of the hepatitis B vaccine in the Brazilian context has been considered decisive in reducing HBV infection rates, even in the presence of risky sexual behaviors [16], although its universal implementation is recent. Nevertheless, the task of discussing growth or reduction in hepatitis B vaccine coverage is still difficult in the Brazilian context, since there are no studies that define the country’s situation in relation to infection and/or vaccination status among MSM.

A number of intrinsic factors may be associated with increased vulnerability of MSM to hepatitis B virus infection, such as sociocultural characteristics and sexual behavior, as well as external factors related to difficulty accessing health services, denial of basic LGBT health rights and institutionalized homophobia [17].

As an example, low proportions of hepatitis B vaccination were reported by younger MSM, even though this population has more access to information than the adult population [18]. In contrast, those who were over 40 had a higher proportion of hepatitis B vaccination, a finding also noted in other studies involving MSM [3, 18, 19].

The predominance of individuals with higher education levels and a higher rate of complete vaccine series (57.8%) corroborates the higher odds of non-completion in participants with only secondary or elementary education. This would indicate that level of education is related to the search for protection and has a direct impact on the percentage of new cases of hepatitis B [3].

HIV-seropositive individuals and those with a history of other STDs presented a higher vaccination percentage (16.2 and 15.1%, respectively), a result complemented by multivariate analysis, which demonstrated that individuals who were unaware of their HIV status were four times more likely to present an incomplete vaccine series. This finding was also reported by other studies that have investigated this population [20, 21].

The association between completion of the vaccine series and a previous STD diagnosis suggests that in the presence of infection, MSM seek to be vaccinated, since during their visit to a specialized health service, screening refers them for further testing and vaccination [20].

The participants who reported going to health units more frequently had a better vaccination history, compared to those who went occasionally (or rarely), which increases the odds of an incomplete vaccine series. This finding demonstrates the importance of health units being prepared to receive LGBT individuals, particularly at the primary care level, since it is the main gateway to the Unified Health System [22]. This result is further supported by evidence that not seeking information from health professionals increases the odds of non-completion of the vaccine series. This reinforces the argument that a structure that drives away the LGBT community worsens their access to the health system and, consequently, vaccination coverage [23].

Because of the growth of STDs among vulnerable populations [24], initiatives and interventions must be reformulated to reduce these populations’ exposure to these infections. As this population does not usually attend health services, tailored strategies implemented in places frequented by MSM could significantly improve vaccination rates. The use of technologies, especially geosocial apps, could serve as a channel to access the MSM population and disseminate information and health services for their use [25, 26].

Our study shows that the factors associated with completion of the hepatitis B vaccine series included social, individual, and programmatic (healthcare) vulnerabilities [27]. Thus, our study provides unprecedented results among MSM, more specifically among users of geosocial dating apps, in relation to vaccination status.

## Limitations

The findings of this study are subject to limitations. The main limitation of this study stands on the fact that it was based on self-reported information by the participants. This information is subject to memory bias, in the absence of vaccination records for verification. To mitigate this limitation, the participants were instructed to consult their vaccination records.

Additionally, the three-dose schedule of the anti-hepatitis B vaccine over a period of months may make it more difficult to remember than other single dose vaccines [28].

Last, it is not possible to generalize these findings to the entire Brazilian MSM population, since the interviewees represented a sample of individuals who used a specific technology for finding sexual partners.

## Conclusion

Completion of the hepatitis B vaccine series was low among men who have sex with men and use geosocial dating apps in Brazil. The factors associated with non-completion were level of education, identification as bisexual, HIV serological status, use of the Internet and health professionals as sources of health information, and frequency of use of health services.

The MSM population is classified as vulnerable to sexually transmitted diseases, since they are more exposed to these infections, including HBV infection. This underscores the need for studies at the national level that address this problem and are able to help provide a clearer overview of the health status (physical and mental) of this population. It is recommended that future studies focus on the use of mobile apps as an environment for campaigns, carrying out interventions through the application itself.

## Abbreviations

CI: Confidence interval
HBV: Hepatitis B virus
HIV: Human Immunodeficiency Virus
LGBT: Lesbian, gay, bi and/or trans
MSM: Men who have sex with men
OR: Odds Ratio
STDs: Sexually transmitted diseases
